# Microsatellite Instability in Colorectal Cancers: Carcinogenesis, Neo-Antigens, Immuno-Resistance and Emerging Therapies

**DOI:** 10.3390/cancers13123063

**Published:** 2021-06-19

**Authors:** Violaine Randrian, Camille Evrard, David Tougeron

**Affiliations:** 1 Gastroenterology and Hepatology Department, Poitiers University Hospital, 86000 Poitiers, France; david.tougeron@chu-poitiers.fr; 2 Faculty of Medicine and Pharmacy, 86000 Poitiers, France; 3 Medical Oncology Department, Poitiers University Hospital, 86000 Poitiers, France; camille.evrard@chu-poitiers.fr

**Keywords:** microsatellite instability, colorectal cancer, deficient mismatch repair, immune checkpoint inhibitor, immunotherapy, microbiota

## Abstract

**Simple Summary:**

A deficient mismatch repair system (dMMR) results in microsatellite instability (MSI). The MSI status of a tumor predicts the response to immune checkpoint inhibitors (ICI) that are now approved in patients with dMMR/MSI metastatic colorectal cancers. In addition to the mechanisms through which MSI can activate the immune system via particular neo-antigens, this review reports the clinical and pre-clinical strategies being developed in the case of resistance to ICI, including emerging therapies and new biomarkers.

**Abstract:**

A defect in the DNA repair system through a deficient mismatch repair system (dMMR) leads to microsatellite instability (MSI). Microsatellites are located in both coding and non-coding sequences and dMMR/MSI tumors are associated with a high mutation burden. Some of these mutations occur in coding sequences and lead to the production of neo-antigens able to trigger an anti-tumoral immune response. This explains why non-metastatic MSI tumors are associated with high immune infiltrates and good prognosis. Metastatic MSI tumors result from tumor escape to the immune system and are associated with poor prognosis and chemoresistance. Consequently, immune checkpoint inhibitors (ICI) are highly effective and have recently been approved in dMMR/MSI metastatic colorectal cancers (mCRC). Nevertheless, some patients with dMMR/MSI mCRC have primary or secondary resistance to ICI. This review details carcinogenesis and the mechanisms through which MSI can activate the immune system. After which, we discuss mechanistic hypotheses in an attempt to explain primary and secondary resistances to ICI and emerging strategies being developed to overcome this phenomenon by targeting other immune checkpoints or through vaccination and modification of microbiota.

## 1. Introduction

The therapeutic impact of a deficient mismatch repair system (dMMR) leading to microsatellite instability (MSI) in colorectal cancers (CRCs) illustrates the importance of deciphering carcinogenesis at both the cellular and the molecular stages. Improved knowledge of the molecular mechanisms involved in cancers facilitates personalized medicine in CRC.

**Figure 1:**
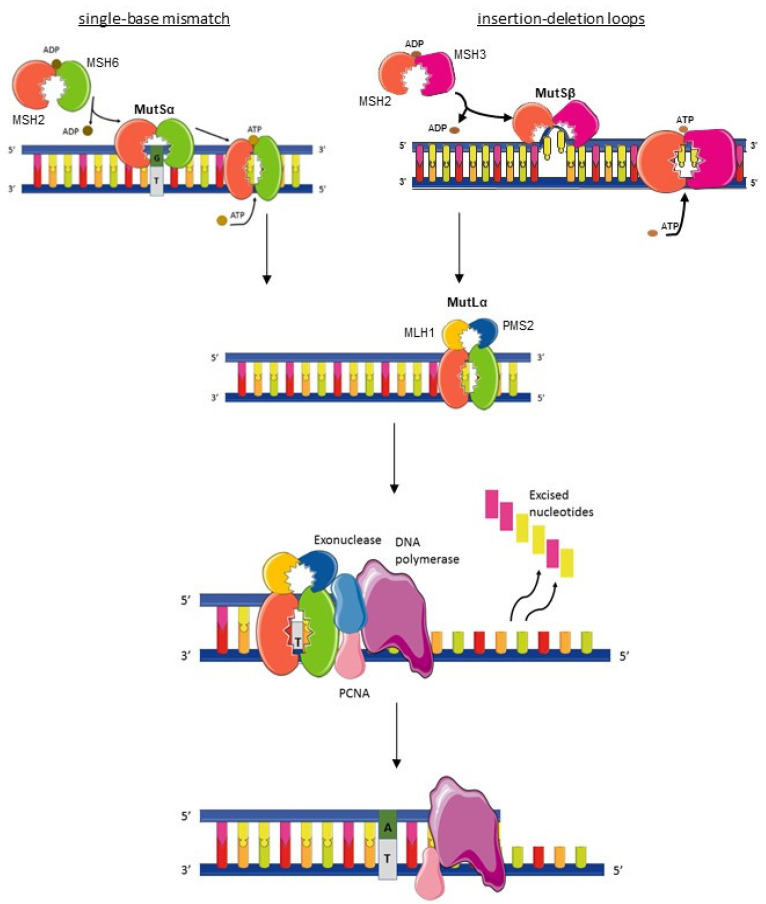
Mismatch repair mechanism. The MutSα (MSH2-MSH6 dimer) recognizes single-base-pair mismatch then surrounds the DNA like a clamp and the MutLα complex (MLH1-PMS2 dimer) is fixed on. The complex MutSβ (MSH2-MSH3 dimer) recognizes insertion–deletion loops, then the MutLα complex is fixed on. Different enzymes (Exonuclease, PCNA, and DNA polymerase) then intervene to excise the mismatched base and to synthesize a new strand of DNA. PCNA: Proliferating cell nuclear antigen. ADP: Adenosine diphosphate. ATP: Adenosine triphosphate.

The proficient mismatch repair system (pMMR), which maintains microsatellite stability (MSS), is defined by the presence of functional proteins involved in this deoxyribonucleic acid (DNA) repair machinery: MutS protein homologue 2 (MSH2), MutS protein homologue 3 (MSH3), MutS protein homologue 6 (MSH6), Human mutL protein homologue 1 (MLH1), post-meiotic segregation increased homologue 1 (PMS1), and PMS1 homologue 2 (PMS2). These proteins work in dimers, MSH2 with MSH6 or MSH3 and MLH1 with PMS2, to scan DNA and to identify base–base mismatches (A-C or A-G instead of A-T) and insertion/deletion loops [1,2] (Figure 1). MSH6 and MSH2 form the MutSα complex, which identifies single-base mismatches. Upon single-base mismatch recognition by MutSα, the heterodimer binds to the MutLα complex formed by MLH1 and PMS2 [3,4]. MutLα interacts with the MutS complexes via MLH1, and this interaction is essential for the coordination of downstream repair events. The proliferating cellular nuclear antigen (PCNA) is involved in MMR initiation as it interacts with MutSα and MutSβ [5]. It also activates MutLα whose endonuclease activity is required for mismatched base excision by the exonuclease I [3,5]. Then polymerase δ, assisted by PCNA, can perform re-synthesis of DNA, and a new DNA strand is sealed by DNA ligase I. Insertion–deletion loops are recognized by the MutSβ complex from by the MSH2-MSH3 heterodimer. MutSβ binds to MutLα to repair the mismatches using the same process as a single-base mismatch [6,7]. The resulting mutation rate is about 2 × 10^−10^ substitutions per base per cell division in the case of a proficient MMR system [8].

Three pathways drive CRCs carcinogenesis: Chromosomal instability (CIN), which accounts for 75% of CRCs; while epigenetic modification of DNA methylation, also called the CpG island methylator phenotype (CIMP), accounts for 20% of CRCs [2]. The last one, dMMR/MSI, occurs in 15% of CRCs and frequently overlaps with CIMP (about 60–80%) [2,6,7]. CIMP can lead to dMMR/MSI status as methylation of CpG islands in the promoter of *MLH1* leads to MLH1 transcriptional silencing. This epigenetic mechanism explains the majority (80%) [2,9,10] of sporadic dMMR/MSI CRCs, which represent more than 80% of diagnosed dMMR/MSI CRCs [5]. By contrast, dMMR/MSI CRC associated with Lynch syndrome (LS) due to a germline mutation of mismatch repair (MMR) genes is not associated with CIMP.

Colorectal carcinogenesis is described as an adenoma–carcinoma sequence. In the CIN pathway, the first genetic events, primarily *AP*C mutation, lead to aberrant colonic crypts and then to adenoma formation [11]. In the MSI pathway and the CIMP pathway, genetic alterations preferentially lead to serrated polyps, which have been described as “flat adenomas” [12,13]. *BRAF^V600E^* mutation is present in about 8% of metastatic CRCs (mCRCs), 30–40% of dMMR/MSI CRCs [14], and 60% of sporadic dMMR/MSI CRCs with *MLH1* promoter hypermethylation [15]. *BRAF^V600E^* mutation is of bad prognosis, independently of the dMMR/MSI status of the CRC [16,17]. However, in some publications, this mutation is no longer a poor prognostic factor for patients with dMMR/MSI CRC [17]. Concerning immunotherapy efficacy in dMMR/MSI mCRC, there is no difference in response according to this *BRAF* mutation [18].

A germline mutation can occur in one of the genes involved in the MMR system defining LS and represents 3% of CRCs and 20% of dMMR/MSI CRCs. While patients with LS have a cumulative risk of developing CRC of about 40-60% at 70 years old, this rate has been lowered with adequate endoscopic follow-up [19]. LS was formerly called hereditary non-polyposis colorectal cancer (HNPCC), but this genetic predisposition includes a large spectrum of cancers: endometrium, ovaries, urinary tract, biliary tract, gallbladder, stomach, small bowel, and skin sebaceous tumors. Among these germline mutations, some directly affect the MMR system, mostly *MLH1*, then *MSH6* and *PMS2* genes. Some mutations affect other genes, for instance a deletion in the *epithelial-cell adhesion molecule* (*EpCAM*) gene silences *MSH2* [20]. The biallelic germline mutation of one of the MMR gene defines the specific entity of constitutional MMR deficiency syndrome (CMMRD), leading to early childhood multiple neoplasia [21].

dMMR/MSI status impacts patient prognosis at all CRC stages as reviewed by Jin et al. [22]. In non-metastatic CRCs, dMMR/MSI is more frequent and is associated with longer disease-free survival (DFS) and longer overall survival (OS), especially in stage II CRCs. Data are more controversial in patients with stage III cancers [23]. In mCRCs, dMMR/MSI does not lead to a better prognosis but rather to shorter PFS than pMMR/MSS mCRCs [24,25]. Incidence of MSI tumors is lower at the metastatic stage suggesting that MSI tumors are usually controlled by the immune system to prevent metastases.

## 2. Consequences of Mismatch Repair Deficiency

### 2.1. Microsatellite Instability

MMR deficiency particularly affects microsatellites, i.e., short-tandem repeats, which are coding or non-coding sequences of repeats of one or up to six bases, up to 60 times. These sequences tend to be misreplicated by DNA polymerases, either through the insertion or deletion of bases. The MMR system is necessary to maintain the stability of these sequences throughout consecutive replications so as to prevent numerous frameshift mutations. The identification of a high number of misreplications (accumulation of insertions or deletions) in microsatellites establishes the diagnosis of MSI [26] and confirms dMMR status. Immunohistochemistry of the four MMR proteins (MLH1, PMS2, MSH2, and MSH6) can detect the dMMR phenotype by nuclear loss of one or more MMR proteins in the tumor tissue. Discrepancies between MSI status and dMMR status exist even though both tests have high sensitivity and specificity. It is a rare phenomenon (1 to 2%), and most “false” discrepancies are due to common errors, for example poor quality of tissue sampling and/or poor quality of tissue fixation and staining [7,27]. Nevertheless, “true” discrepancies have been described when an inactive mutated MMR protein could remain expressed (missense mutation) but with a loss of function leading to a pMMR/MSI tumor [28].

### 2.2. Tumor Mutational Burden

dMMR/MSI status dramatically increases tumor mutational burden [27,29]. In a study comparing the sequenced DNA of ten dMMR cancers with nine pMMR cancers (both colorectal and non-colorectal cancers), dMMR status resulted in a mean of 1782 somatic mutations per tumor as compared to 73 mutations per tumor in pMMR cancers [30]. Besides, in the analysis of 100,000 cancer genomes [31,32], 97% of MSI tumors had more than 10 mutations/Megabase (Mb) compared with the median of 4.5 mutations/Mb in the 7758 all-comer CRCs (including both pMMR/MSS and dMMR/MSI). MSI and TMB mostly [33,34] but only partially overlap, as a small fraction of mCRCs with high TMB are MSS (about 0.5 to 3%), while other DNA repair systems can be deficient [35]. For instance, somatic or constitutional mutations of *DNA polymerase delta* (*POLD*) and *epsilon* (*POLE*) generate numerous mutations leading to high TMB [35,36]. Somatic *POLE* mutations are present in a small fraction of CRCs (1-2%) [28] and *POLD* mutations are extremely rare in CRCs [31]. Germline mutations of *POLE* associate polymerase proofreading-associated polyposis syndrome (PPAP) with an increased risk of adenomas, CRCs, and extra-gastrointestinal tumors (ovary, endometrium, brain). In addition, DNA repair genes such as *ATR, FANCM, PRKDC, POLD1, SMARCA4, TLK1, XPC,* and *MRE11A* [30] were found mutated in dMMR/MSI tumors, which increases TMB. Finally, a high TMB seems to be an efficient biomarker to predict response to ICI in different kind of tumors including dMMR/MSI CRCs and could be used as predictor for future trials in these tumors [30,37]. Nevertheless, in dMMR/MSI mCRC, it remains to be demonstrated that tumors with low TMB are those that do not respond to ICI. In addition, perhaps other biomarkers like immune scores or neo-antigen load are more relevant.

### 2.3. Microsatellite insTability Target Genes

Most mutations in microsatellites lead to frameshift mutations. A frameshift mutation results from an insertion or deletion of a number of nucleotides that is not a multiple of three, then alters the amino-acid sequence and produces truncated proteins that are frequently non-functional. The first MSI target gene identified is involved in cell proliferation, and the frameshift in the *tumor growth factor β receptor II* (*TGFβ-RII*) leads to a loss of function (Figure 1) [38]. This frameshift mutation occurs frequently in dMMR/MSI CRCs (80%) [37].

Other genes involved in carcinogenesis were identified as MSI target genes. Indeed, pro-apoptotic factors are recurrent MSI target genes: *FAS, BAX, Bcl10,* and *Caspase 5* were found mutated in 62%, 51%, 13%, and 10% of dMMR/MSI CRCs [39,40]. Genes involved in cell cycles such as *PTEN* [40], and tumor suppressor genes such as *AIM2* [41], as well as genes involved in DNA repair—h*MSH6*, h*MSH3, ATR, PRKDC, MBD4* [39,40]—have also been identified.

**Figure 2:**
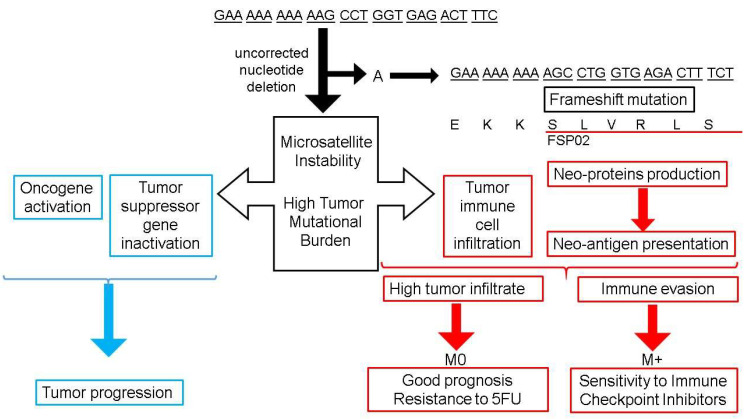
Mismatch Repair deficiency: Consequences on tumor biology and outcome in colorectal cancer. The upper right part of the figure shows an example of frameshift mutation in the *tumor growth factor β receptor II* (*TGFβ-RII*) gene. The single nucleotide deletion of an adenosine (A) is not corrected by a deficient mismatch repair, leading to a frameshift mutation and a modified sequence of amino-acids corresponding to the frameshift peptide (FSP02). The left part of the figure summarizes the consequence of frameshift mutation on tumor progression. The lower part of the figure summarizes consequences of such uncorrected mutations on immune tumor environment and their clinical impact. M0: Non metastatic colorectal cancer; M+: Metastatic colorectal cancer.

It is remarkable that MSI not only inactivates target genes but also, by disrupting the open reading frame, can result in the appearance of neopeptides, some of them being potentially immunogenic. This has been shown, in particular, for *TGFB-RII*, which harbors a 10-base pair (bp) polyadenine tract (Figure 2) [38]. In addition, excess TGF-β due to the *TGFB-RII* mutation that cannot bind to its receptor could promote the differentiation of regulatory T lymphocytes [42].

### 2.4. miRNA Modifications

When affecting non-coding sequences, MSI status can alter micro-ribonucleotid amino-acids (miRNAs) with a significant impact, as some miRNAs are involved in gene regulation. Slattery and colleagues showed that MSI status impacts miRNAs production [43]. Among 1893 individuals with CRC whose miRNAs were assessed, 170 tumors were diagnosed MSI (9%). Among the set of 94 miRNAs that were differentially expressed between the 170 MSI tumors and their MSS counterparts, 91% were downregulated. These differentially expressed miRNAs were involved in signaling pathways including cell cycle regulation, *PI3K/AKT*-signaling, *PTEN*-signaling, *TP53*-signaling, *IGF-1*-signaling, and *TGFβ*-signaling. This large study confirmed these previous reports, showing differential expression of miRNAs involved in cell cycle, DNA replication, DNA recombination, and DNA repair between MSI and MSS CRCs on smaller series [44]. Despite these molecular implications, miRNAs expression did not appear as a prognostic factor in MSI or MSS CRCs. Today, miRNA are not biomarkers used routinely for CRC management due to the limited prognostic value and the few studies available in CRCs.

### 2.5. Neo-Antigen Production

dMMR/MSI status induced frameshift mutations in coding DNA sequences that can frequently result not only in a truncated non-functional protein, but also in neo-antigen production. Regardless of pMMR/dMMR cancer status, when applying exome data to an epitope prediction algorithm, 30% of mutations were predicted to produce neo-antigens, even though dMMR/MSI CRCs have higher TMB [30].

A pre-clinical study showed that pMMR tumors could be converted to dMMR tumors through genome editing technology [45]. Authors have applied the recently Nobel-awarded technology involving clusters of regularly interspaced short palindromic repeats (CRISPR) to inactivate MLH1 in each cell belonging to a culture of colonic epithelial cells. These edited tumors had a higher TMB than pMMR tumors. Furthermore, they were able to trigger high adaptive immunity as far as they expressed more neo-antigens. Therefore, the authors identified 578 potential mutation-associated neo-antigens in dMMR cancers and only 21 of these neo-antigens in pMMR cancers. Other groups confirmed that MSI status is associated with TMB and high neo-antigen production [30,31,32]. Recently published studies compared sequencing to multiple existing datasets, providing insights into the peptides generated in MSI tumor cells from 338 patients diagnosed with dMMR/MSI CRC (83 tumors), dMMR/MSI endometrial cancer (170 tumors), or dMMR/MSI stomach cancer (85 tumors) [46]. These MSI tumors had a mean frameshift mutation load more than 300 times higher than in MSS tumors. On average, frameshift peptides were 20–30 amino-acids long. Authors were able to show peptide immunogenicity encoding poly-epitopes and binding to a broad spectrum of major histocompatibility complex class I (MHC-I) alleles. Immunogenic peptides were widely shared between dMMR/MSI tumors. Out of the 83 patients with dMMR/MSI CRC, the authors obtained 14 samples of blood mononuclear cells and tested their immunogenicity in vitro. The reactive CD8 T-cell population was twice increased in the case of 11 out of 14 samples. Missense peptides, which were closer to self-peptides, were less frequent and generated fewer epitopes than frameshift peptides. Mutations affecting the Human Leukocyte Antigen (HLA) machinery were reported in 11% of MSI CRCs analyzed and were associated with the immune-resistance of dMMR/MSI tumors [46]. Of note, in this study, the neo-antigen burden correlated with T-cell memory tumor infiltration suggesting that prior neo-antigen tumor presentation and recognition is required to set tumor infiltration by effector T-cells. While this level of neo-antigens probably reflected sensitivity to ICI today, we do not have a validated technique to determine it.

### 2.6. Immune Tumor Infiltration

dMMR/MSI CRCs present a high rate of tumor-infiltrating lymphocytes (TILs) [47]. CD3+ TILs are effector cells of the tumor immune response and express different surface membrane markers depending on their role in the immune response. Regulatory CD3+ CD4+ T-cells express Foxp3 and represent a minor fraction of TILs. In all-comer CRCs, FoxP3 TILs enrichment was associated with poor immune response [48] and poorer prognosis [49]. Regulatory T-cells were shown to have conflicting prognostic effects depending on TGF-β expression: In TGF-β high expressing CRCs, Foxp3 TILs were associated with better prognosis than in their TGF-β low expressing counterparts [50]. When focusing on dMMR/MSI CRCs, Foxp3 TILS were associated with less invasive tumors [51,52]. The majority of TILs were CD3+ CD8+ cytotoxic T-cells, which were able to kill tumor cells [52]. These lymphocytes have been shown to specifically recognize frameshift peptides generated by MSI [53,54]. The neo-antigen load determined by a prediction pipeline applied to whole exome sequencing data correlated with immune tumor infiltration scored upon pathologists’ reading [55]. While TILs from freshly resected dMMR/MSI CRCs were able to kill cells from MSI-derived cell lines, they failed to kill cells from MSS CRC cell lines [56]. In the same study, circulating T lymphocytes isolated from blood samples of LS patients without CRC were also able to target frameshift peptides and perhaps explain the low cancer rate in some LS patients. Immune response is oriented through CD4+ T helper cells (Th) defined by the interleukins they produce. The relevant Th cells in anti-tumor response are Th1, which produce interferon (IFNg), and Th17, which produce interleukin 17 [56] and stimulate CD3+ CD8+ TILs through these signals.

To prevent excessive immune response, some immune checkpoint molecules have been found at the surface membrane of CD8+ TILs. When these molecules, such as programmed death 1 (PD1), interact with their ligand programmed death-ligand 1 (PD-L1) on antigen-presenting cells, tumor cells, Th cells, or macrophages, the CD8+ T-cells remain inactive. A set of immune checkpoints can downregulate T-cell response: Cytotoxic T-lymphocyte-associated protein 4 (CTLA4), T-cell immunoreceptor with immunoglobulin and immunoreceptor tyrosine-based inhibitory motif (ITIM) domains (TIGIT), Lymphocyte-activation gene 3 (LAG3) [57], V-domain Ig suppressor of T-cell activation (VISTA), T cell immunoglobulin and mucin domain-containing protein 3 (TIM-3), and inducible T-cell co-stimulatory protein (ICOS) [58]. In dMMR/MSI CRCs, PD-L1 overexpression on tumor cells can repress cytotoxic CD8+ TILs proliferation and activation [59]. PD-L1 overexpression was found in only about half of dMMR/MSI cancers. It was located not only on the tumor cells, but also on the membrane of TIL and tumor-associated macrophages. These data suggest that immune checkpoint expression could be used more easily in routine to refine prognosis rather than the description of lymphocyte subpopulations.

## 3. Immune Resistance in dMMR/MSI CRCs

### 3.1. Biomarkers of Response to Immune Checkpoint Inhibitors

Immune checkpoint inhibitors form a recent therapeutic approach. Their efficacy relies on immune infiltration of the tumor, immune checkpoint expression, TMB, and neo-antigen formation. These biomarkers are poorly studied in dMMR/MSI CRCs, and even fewer data about these biomarkers in dMMR/MSI CRCs under ICI treatment are available [59].

A quantification of immune infiltration in all-comer CRC was validated by several studies. Immunoscore© is a validated biomarker to quantify the amount of CD3+ and CD8+ lymphocytes, both at the tumor center and at the invasive margin. It better correlated to prognosis in localized tumors than to the T and N stages [60]. dMMR/MSI status correlated with a high Immunoscore© as 56% of dMMR/MSI CRCs had the maximal Immunoscore© IS4 and only 26% in pMMR/MSS CRCs [61]. Immunoscore© remained an independent prognostic factor among patients with dMMR/MSI tumors with better than 5-year disease-free survival (DFS) and overall survival (OS) for high Immunoscore© [62]. CD4+ and CD8+ TILs in dMMR/MSI CRCs expressed more negative immune checkpoints than their pMMR/MSS counterparts [62].

PD-L1 expression was associated with a poor prognosis whatever the tumor stage, overwhelming the positive impact of the level of TILs due to a state of anergy [54]. The PD1 co-receptor was not the only co-inhibitory immune checkpoint concerned. TIM-3, LAG-3, TIGIT, and ICOS were also more often observed at the CD8+ and CD4+ lymphocytes membrane in dMMR/MSI CRCs and at an early tumoral stage. [63].

Recently, four consensus molecular subtypes (CMS) with distinguishing features were identified in CRC, CMS 1 (immune, ~15%), CMS 2 (canonical, ~35%), CMS 3 (metabolic, ~15%), and CMS 4 (mesenchymal, ~25%) [64]. CMS1 and CMS3 concerned MSI CRCs and CMS2 and CMS4 were canonical subgroups with high somatic copy number alterations, without microsatellite instability. MSI status in localized CRCs was mainly associated with high TMB, immune activation, and infiltration, which was defined as CMS 1. This status was associated with less recurrence in non-metastatic CRCs but poorer OS after relapse. Immune evasion with proliferation of sub-clones uncontrolled by the immune system could account for this poor prognosis after relapse and/or at a metastatic stage. A small fraction of MSI CRCs was classified as CMS3, that is to say without high TMB or high immune infiltration. This minor subset of MSI CRCs remains under-investigated but perhaps explains 30-40% of the dMMR/MSI mCRCs resistance to ICI [18,65]. TMB was recently shown to be predictive of the response to ICI in dMMR/MSI CRCs [37].

Up until now, the load of frameshift peptides has not been identified as an independent prognostic factor in dMMR/MSI CRCs [46]. This result is disturbing since the higher the number of frameshift peptides, the higher the number of antigens, immune infiltrates increase, and prognosis should be better. Nevertheless, many mechanisms of immune escape have been identified in dMMR/MSI CRCs and can explain this absence of correlation between the number of frameshift peptides and prognosis.

Upcoming clinical trials including ancillary studies with these different biomarkers would be very helpful to determine the most robust parameters to predict the response to ICI in dMMR/MSI CRCs.

### 3.2. Alteration of Antigen Presentation Machinery

CRCs with a hypermutated phenotype, which partially overlap with dMMR/MSI status, have multiple mutations affecting their human leukocyte antigen (HLA) machinery [66]. HLA class 2 presentation was absent in one-third of dMMR/MSI CRCs. The HLA class 2 regulatory gene RFX5 was identified as mutated due to microsatellite alterations [67]. HLA class 1 loss was more frequent in MSI CRCs (60.6%) than in MSS CRCs (16.7%; *p* < 0.0001) [68]. Mutations in Beta-2-microglobulin (B2M) or the antigen presentation machinery gene such as transporter-associated with antigen processing (TAP) were involved in the absence of frameshift peptide presentation [64]. B2M loss was shown to occur in about 10% of dMMR/MSI CRC [68,69]. This loss had also been shown by other groups to confer primary resistance to anti-PD1 therapy in patients treated for melanoma [70], or dMMR/MSI CRC [71].

Ballhausen and colleagues collected tissue from CRC and endometrial MSI cancers and studied the immunogenicity of neo-antigens derived from coding microsatellites in *B2M*-wild-type tumors and *B2M*-mutant tumors [72]. They showed that in *B2M*-wild-type MSI tumors, immunoediting was at work as the cellular clones selected upon cancer evolution contained neo-antigens derived from coding microsatellites with low predicted immunogenicity. In *B2M*-mutant tumors, there was no active immunoediting, that is to say no adaptation of the immune repertoire toward the existing tumor. The authors suggested that as HLA class I antigen presentation was impaired, MSI cell clones containing coding microsatellites with neo-antigens with high predicted immunogenicity were therefore not cleared by T-cells. These data support the hypothesis that in *B2M*-mutant tumors, even those with an active adaptive immune response, immune escape can occur through antigen presentation impairment. This immune evasion model, due to antigen presentation machinery mutations, strongly supports the hypothesis previously proposed by Giannakis and colleagues and accrued data on *HLA* and *B2M* mutation frequency in dMMR/MSI tumors [69,73,74].

### 3.3. Inefficient Tumor Cell Lysis

Other mechanisms in the alteration of the antigen presentation machinery could be involved in the resistance to ICI. For instance, the loss of FAS signaling allows tumor cell evasion from CD8+ T-cell lysis [75]. Gurjao and colleagues thoroughly analyzed the tumor of one of the dMMR/MSI mCRC patients whose disease had primary resistance to the anti-PD1 Pembrolizumab [76]. They confirmed the dMMR/MSI status of the tumor with a high TMB. Single-cell analysis showed that the tumor was infiltrated not only with Natural Killer (NK) cells, but also with immunosuppressive M2-polarized macrophages. The high density of NK cells at the center of the tumor first appeared inconsistent with tumor resistance to ICI, as NK cells can perform tumor cell lysis without antigen presentation. Indeed, further analysis showed antigen presentation impairment, suggesting that antigen presentation is required to educate NK cells: NK cells perform the work of cell lysis correctly but with an incorrect presentation of neo-antigens making this lysis ineffective in terms of tumor control. This phenomenon, combined with M2-polarized macrophages, which can prevent NK cell degranulation [77], accounted for NK cells’ inefficiency and primary resistance to anti-PD1.

### 3.4. Impaired DNA Sensing

MLH1 regulates Exonuclease 1 excision activity [78]. It was lately shown that MLH1 deficiency results in hyperexcision of DNA and accumulation of cytosolic DNA. This phenomenon activates the cytosolic DNA sensor cyclic GMP-AMP synthase (cGAS), an important player in innate immunity, which is able to activate the adaptor stimulator of interferon genes (STING) [78]. STING activates the Interferon pathway, which in turn triggers dendritic cells presentation and stimulation of cytotoxic CD8+ T cells [79]. As shown in animal models, cGAS is indispensable to the immune response to ICI [80]. Furthermore, high cGAS expression appeared as a prognostic factor, associated with prolonged disease-free survival and overall survival in dMMR/MSI tumors [81]. These data propose the cGAS-STING pathway as an important pathway to enhance response to ICI in patients with dMMR/MSI tumors. Further basic and clinical studies are needed to support this hypothesis and to efficiently target this pathway.

## 4. Immune Activation and Microbiota in dMMR/MSI Colorectal Cancers

**Figure 3:**
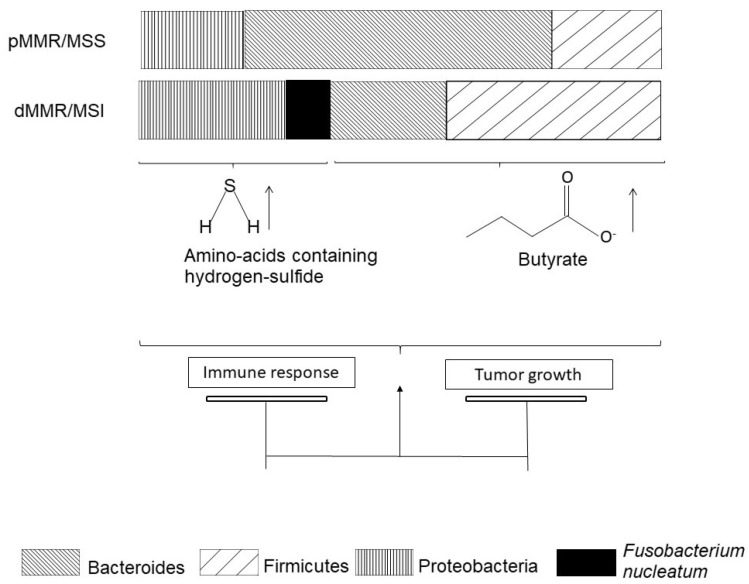
pMMR/MSS colorectal cancers and dMMR/MSI colorectal cancers have different microbiota.

Microbiota from patients with CRC significantly differ from the microbiota of healthy people [82]. Indeed, Bacteroides and Firmicutes are enriched in the microbiota of CRC patients (Figure 3). Among the species identified, Fusobacterium nucleatum is highly specific to primary and metastatic tumors [83]. Lee JA and colleagues searched for *Fusobacterium nucleatum* DNA in tumors from 126 patients diagnosed with dMMR/MSI CRC [84]. They were associated with more M2-polarized macrophages [85] at the center of the tumor. *Fusobacterium nucleatum* was also associated with MSI status in a Japanese population with CRCs [86]. All-comer adenomas were enriched with *F. nucleatum* as compared to normal tissue [87]. Mice fed with *F. nucleatum* were enriched with myeloid-derived suppressor cells (MDSCs), which efficiently suppressed T-cell activity [87]. Data available in a series of 598 all-comer CRCs confirmed the correlation of MSI status with *F. nucleatum* enrichment, which inversely correlated with CD3+ T lymphocyte infiltration [87]. The correlation of *F. nucleatum* enrichment with FoxP3 TILs remains unclear [84,87,88]. These data suggest that MSI status drives *F. nucleatum* enrichment in patients with dMMR/MSI CRCs, which might accelerate colorectal tumorigenesis.

Beyond species description, the community has grown interested in the metabolomic impact of microbiota alterations (Figure 3). Hale [89] and colleagues compared microbiota from 25 patients with MSI CRC to microbiota from 58 patients with MSS CRC. They reported that MSI/MSS status is the strongest factor inducing consistent variations in gut microbiota. Several *Bacteroides* and *Fusobacterium* species, especially *F. nucleatum*, were associated with MSI CRC and promoted production of amino acids containing hydrogen-sulfide (Figure 2). As the authors discuss, hydrogen-sulfide is thought to have a dose-dependent role in colon cancer carcinogenesis: tumor promotion at low doses [90] and tumor suppression at high doses [91].

Post-operative analysis of microbiota in LS with either colorectal, endometrial, or ovarian cancers showed enrichment in *Bacteroides* species [92]. Pre-clinical models of LS suggested the implication of gut microbes in dMMR/MSI CRC carcinogenesis through epigenetic downregulation of *MSH*2 by metabolites such as butyrate. The transcriptional silencing of *MSH2* ended up with MSH2^−/−^ colonic cells that were therefore turned into dMMR/MSI cells, enhancing their proliferation and transformation into cancer cells [93]. In this study, the epigenetically obtained MSH2^−/−^ mice displayed many polyps in the small intestine and colon. Some animals received an antibiotic cocktail *in utero* and up to six weeks of age. Upon this treatment, the number of polyps they displayed at six weeks old exhibited a two to six-fold decrease. The antibiotic-fed MSH2^−/−^ mice receiving butyrate instillation displayed an increased number of polyps. In healthy LS patients, fecal microbiota displayed a significant increase of *Bacteroidetes* and *Proteobacteria* and a significant decrease of *Firmicutes* [94]. Further insights in microbiota composition in LS patients are required to set up interventions to modify microbiota in LS to prevent CRC.

## 5. Overcoming Resistance to Immunotherapy 

As discussed above, dMMR/MSI CRCs are characterized not only by strong immunogenicity and dense immune tumor infiltration, but also by mechanisms of resistance to allow tumor progression that are identified as specific to dMMR/MSI CRCs. We will now discuss the therapeutics developed to overcome this immune-resistance (Table 1).

The anti-PD1 Pembrolizumab was shown to increase survival in dMMR/MSI mCRC patients in the Keynote 177 study. In first-line metastatic setting, PFS was 16.5 months with Pembrolizumab compared to 8.2 months with chemotherapy [65]. However, a subgroup of about 35% of patients with dMMR/MSI mCRC did not respond to ICI and rapidly experienced progression upon treatment with Pembrolizumab. Predictive factors of this primary resistance or hyperprogression are still unknown. These phenomenon need to be distinguished from pseudo-progression, which corresponds to a strong and rapid inflammatory response followed by tumor shrinkage. In addition, about 30% of dMMR/MSI mCRC patients treated with Pembrolizumab have secondary resistance to ICI [65].

### 5.1. Combining Immune Checkpoints

In the CheckMate 142 study [18], patients with dMMR/MSI mCRC received anti-PD1 Nivolumab. Similar to Keynote 177, 30% of patients experienced early progression. To overcome primary resistance to anti-PD1, Overman MJ and colleagues [95] proposed synergic use of anti-PD1 and anti-CTLA-4, combining Nivolumab and Ipilimumab every three weeks (Table 1). In this phase II trial, among the 119 patients enrolled, only about 15% presented primary resistance at the 3-month evaluation [95]. Although these trials were not designed to be compared with one another, the authors suggested in the discussion section that the tested combination of ICI divided by two was the rate of primary resistance to ICI observed under monotherapy. The disease control rate at 12 weeks was 80% with the combination of Nivolumab and Ipilimumab versus 69.8% with Nivolumab alone. Secondary resistance also seemed lower, but patients under a combination of ICI presented higher rates of grade 3 adverse events (27% versus 18%) and grade 4 adverse events (5% versus 3%). This combined strategy is still under investigation in several trials. A phase II trial is testing Pembrolizumab combined with the anti-CTLA4 MK1308A in dMMR/MSI mCRC (NCT03179436) and another trial is testing the anti-PD1 Sintilimab in combination with an anti-CTLA4, IBI310, as well as in dMMR/MSI metastatic or locally advanced CRC (NCT04258111).

### 5.2. New Immune Checkpoint Inhibitors and Combination to Targeted Therapies

Immune checkpoint molecules were found to be significantly more expressed in dMMR/MSI CRCs than in pMMR/MSS CRCs [96]. Therefore, combinations of monoclonal antibodies against LAG3, TIGIT, VISTA [97,98], PD-1, PD-L1, and CTLA-4 should help to circumvent primary resistance of dMMR/MSI mCRCs to Pembrolizumab. Ongoing clinical trials are testing molecules targeting LAG3 [99], whose ligand is MHC II. Among them, some trials are enrolling patients with dMMR/MSI CRC (NCT02060188) treated by anti-LAG3 molecule combined with anti-PD1/PD-L1. A trial testing different combinations of Nivolumab, Ipilimumab, and OX40 (CD134) agonist in advanced solid tumors, mainly NSCLC and bladder cancers, [100] did not show improved results as compared to Nivolumab combined to Ipilimumab.

Some targeted therapies are combined with ICI such as the anti-PD1 Camrelizumab with the vascular endothelial growth factor inhibitor Apatinib in locally advanced dMMR/MSI CRCs (NCT04715633). Indoleamine 2,3 dioxygenase 1 (IDO1), which is involved in tryptophan metabolism, was associated with tumor infiltration modulation towards a tolerant response by recruiting and activating both myeloid-derived suppressive cells (MDSC) and Tregs [101]. In a phase I study [102], anti-IDO1 treatment was well-tolerated. In patients with CRCs, 4/5 patients underwent progression within two months of treatment and one at six months. Unfortunately, MSI/MSS status was not available. Frequent IDO increase in dMMR/MSI CRCs (28.1%) [57] suggests it could be targeted in association with ICI.

### 5.3. Boosting Immunotherapy with Microbiota

The response to anti-CTLA4 treatments depends on *Bacteroides* species as shown both in mice and patients with melanoma and non-small cell lung cancer (NSCLC) [103]. Antibiotic consumption was analyzed in patients diagnosed with various carcinomas and treated with ICIs (anti-PD1/anti-PD-L1 and/or anti-CTLA4). Patients with no antibiotic treatment two months before or one month after ICI initiation exhibited longer OS (15.3 months) than patients who had received antibiotics within this period (8.3 months) [104]. Moreover, fecal microbiota composition was predictive of the response to anti-PD1 therapy. *Akkermansia muciniphila* was enriched in microbiota of patients with better prognosis in NSCLC or renal cell carcinoma (RCC). *Akkermansia muciniphila* was sufficient to restore anti-PD1 response in mice transplanted with feces from non-responder patients [104]. As in all studies on immunotherapy and microbiota in CRC or other tumor types published so far, there were no details about the MSS/MSI or pMMR/dMMR status of tumors. Other groups showed that abundant *Bifidobacterium longum*, *Collinsella aerofaciens*, and *Enterococcus faecium* [105] or members of *Ruminococcaceae* [106] in microbiota were associated with better clinical response to ICI in melanoma patients. The first human trials testing fecal microbiota transplant (FMT) from melanoma patients with ICI response to patients with progression on anti-PD1 therapy showed encouraging results [107,108]. In one study, two partial responses and one complete response out of ten patients were observed [107]. A single FMT from an ICI responder modified gut microbiota of ICI-primary resistant patient and could induce response [108].

A pre-clinical study showed a direct interaction in human cell lines between *F. nucleatum* and TIGIT, suggesting that antibiotic treatment targeting *F. nucleatum* could enhance the response to anti-TIGIT therapy [109]. *Bifidobacterium longum* is already being tested in combination with Pembrolizumab in phase I/II, enrolling patients with MSI advanced tumors (NCT03775850). Further analyses are required to identify the species specifically associated with response to ICI in patients treated for dMMR/MSI mCRCs to modulate microbiota and/or to evaluate fecal microbiota transplant.

### 5.4. Vaccination with Frameshift Peptides

Some studies showed that frameshift neoantigens were more numerous in dMMR/MSI cancer patients responding to anti-PD1 [46]. These data suggest that a vaccine strategy designed to enhance frameshift-peptide production in patients with primary or secondary resistance to ICI could be successful. Kloor and colleagues [110] enrolled patients with dMMR/MSI mCRC in a phase I/II trial testing subcutaneous vaccination with selected immunogenic frameshift neo-antigens. This treatment delivered in pre-treated patients (i.e., first, second, and third line of palliative standard therapy) was well-tolerated and could induce humoral and cellular immune responses in all 22 patients enrolled in the study. One heavily pretreated patient with bulky metastases showed stable disease over 7 months. A phase I trial with a vaccine encoding various frameshift peptides shared among MSI tumors is ongoing (NCT04041310) [111]. Vaccines could be combined with ICI such as proposed in phase I KEYNOTE-603 (NCT03313778) testing mRNA-4157 with Pembrolizumab with an acceptable tolerance and 8 out 20 patients experienced disease control. This trial included patients with dMMR/MSI tumors; unfortunately, subgroup results were not presented.

### 5.5. Targeting the Interferon Signaling Pathway

Genomes of tumors with primary resistance to ICI such as melanoma and dMMR/MSI CRCs were sequenced [112]. The loss of Janus kinase (JAK1/2) function was identified to downregulate interferon production signaling. These JAK1/2 mutations were associated with PD-L1 loss of expression at the tumor cell membrane and could explain resistance to anti-PD1/PD-L1. When introducing wild-type *JAK1/2* in mutant cell lines, interferon production was followed by the acquisition of PD-L1 at the cell membrane [112]. *JAK1* and *JAK2* mutations were encountered in 10% and 5% of all-comer CRCs, but *JAK1* mutation was found in 18% of dMMR/MSI CRCs [112]. *JAK1*-mutated dMMR/MSI CRCs presented lower expression of PD-L1 and downregulated signaling of both interferon and PD1 signaling. *JAK1* mutations have not been found to be associated with poor prognosis, but rather with impaired antigen presentation and the promotion of immune evasion [113].

## 6. Conclusions

Due to a high burden of neo-antigens derived from frameshift mutations, an immune response is efficient in most dMMR/MSI CRCs conferring a good prognosis of these tumors at an early stage of the disease. At the metastatic stage, in about 70% of patients, this immune response is efficiently enhanced by ICI, which are now approved as a treatment of first-line dMMR/MSI mCRCs. Immune evasion is mostly due to altered antigen presentation machinery and/or overexpression of negative immune checkpoints and explains most primary and secondary resistances to ICI. Consequently, new ICI alone or in combination with other drugs are emerging modalities for treating dMMR/MSI CRCs. Gut bacterial modulation or fecal microbiota transplant could stimulate the immune response in patients with dMMR/MSI CRCs with secondary resistance to ICI. Microbiota modulation and increased antigen presentation appear as the two arms through which to activate new therapeutic strategies aimed at restoring efficient immune response and ICI efficacy in dMMR/MSI tumors.

## Figures and Tables

**Table 1:** Therapeutic development to overcome resistance to immune checkpoint inhibitors in dMMR/MSI CRCs.

Reference/Trial	Type of ICI/Target	Name of Drug	Phase
86	Anti-PD1 and anti-CTLA-4	Nivolumab and Ipilimumab	II
88, 89, NCT04475523	Anti-VISTA	CI-8993	I
90, NCT03607890	Anti-LAG3	Relatlimab	II
NCT04715633	Anti-PD1 with vascular endothelial growth factor inhibitor	Camrelizumab and Apatinib	II
92 and 93	Anti-IDO1	Navoximod	I
99 and 100	Gut Microbiota	Fecal microbiota transplant	I
102 NCT04041310	Vaccines with MSI-shared frameshift peptides	*TAF1B*, *HT001*, *AIM2* in Micoryx GAd20-209-FSP and MVA-209-FSP	I/IIa I
104, 105, NCT04303403, NCT02955940	Janus kinase (JAK1/2)	Ruxolitinib	I, II

FSP: Frameshift peptide, ICI: Immune checkpoint inhibitor, PD1: Program death 1, VISTA: V-domain Ig suppressor of T cell activation, LAG3: Lymphocyte activation gene-3, IDO1: Indoleamine 2,3 dioxygenase 1, JAK 1-2: Janus kinase 1 and 2.
